# Assessing visual performance during intense luminance changes in virtual reality

**DOI:** 10.1016/j.heliyon.2024.e40349

**Published:** 2024-11-16

**Authors:** Niklas Domdei, Yannick Sauer, Brian Hecox, Alexander Neugebauer, Siegfried Wahl

**Affiliations:** a Carl Zeiss Vision International GmbH, Aalen, Germany; b Bad Ridge Games, Seattle, WA, USA; c Institute for Ophthalmic Research, Eberhard Karls University Tübingen, Tübingen, Germany

## Abstract

During indoor-outdoor transitions humans encounter luminance changes beyond the functional range of the photoreceptors, leaving the individual at risk of overlooking harmful low-contrast objects until adaptation processes re-enable optimal vision. To study human visual performance during intense luminance changes, we propose a virtual reality based simulation platform. After linearization of the headset's luminance output, detection times were recorded for ten participants. The small (FWHM = 0.6°) low-contrast stimuli appeared randomly in one of four corners (±10°) after luminance changes of three magnitudes within 1 or 3 s. Significantly decreased detection times were observed for the conditions with simulated self-tinting lenses compared to lenses with fixed transmission rates after luminance decreases. In cases of luminance increases all detection times were similar. In conclusion, the proposed virtual reality simulation platform allows for studying vision during or after steep luminance changes and helps to design technical aids like self-tinting lenses.

## Highlights

• The HTC Vive provides a practicable luminance range of 3 magnitudes, allowing the simulation of indoor-outdoor transitions.
• Visual performance after steep luminance decreases was significantly better with simulated self-tinting spectacles.
• Different transmission rates of the simulated lenses did not affect the detection times after luminance increases.

## 1 Introduction

The human visual system is functional across an enormous luminance range of about 14 magnitudes [1]. For example, humans can observe a sunny outdoor scene, but also detect individual photons [2,3]. This is possible, first, because of two different photoreceptor cell types in the human retina: the cones and the rods. The rods are operational at scotopic and mesopic light levels, while the cones are functional at high light levels (mesopic and photopic) and enable color vision [4]. Second, adaptation plays an important role. Without adaptation, the individual photoreceptor has a functional range of about 3 magnitudes. The overall photoreceptor activation to luminance relationship follows a sigmoid function and has a central part of 1.5 log10 units, where luminance is linearly correlated with activation level [[5], [6], [7]]. This observation matches closely with the luminance range within a natural scene, spanning approximately 2 magnitudes [1,8].

However, transitions between indoor and outdoor, for example when driving into a tunnel or walking into a building on a sunny day, put the photoreceptor's functional range at its limits, with luminance changes of up to 3 magnitudes [9]. Thus, to enable optimal vision and to perceive low-contrast objects or obstacles, a shift of the photoreceptor's functional range is indispensable and is achieved via adaptation [10]. A rapid (hundreds of milliseconds) adaptation mechanism of the visual system is the adjustment of the pupil diameter. Theoretically, a range of 2–8 mm is possible, thereby allowing adjustment of retinal illuminance by a factor of 16 (or 1.2 log10 units) [11]. Practically, this range only occurs in healthy young adults during transitions from photopic to scotopic illuminance. In the scenario of a young driver entering a tunnel, pupil diameter changes from 4 to 7 mm were reported [12], which changes retinal illuminance by a factor of about 3 (or 0.5 log10 units). For elderly people (>60 years) pupil diameters are decreased, but the range is similar [12]. At the same time, the photoreceptors adapt as well, but this process is rather slow compared to the pupil response: For example, to shift the cone's functional range by 1 magnitude, it takes about 30 s [10]. Taken together, this leaves a significant timescale during indoor-outdoor transitions within which humans are unable to perceive low-contrast objects, resulting in increased visual discomfort [13]. For example, when moving into a significant darker environment on a bright day, like driving into a tunnel or entering a poorly lit building on a sunny day, which do not meet modern standards.

Such troublesome scenario can be tackled by sophisticated lighting in modern architecture [14]. Or via specialized eye-wear like self-tinting lenses, which can switch between a low transmission (“tinted”) state, blocking most of the light in a high luminance environment like outdoors, and a high transmission (“clear”) state in a low luminance environment. Because of this, the encountered luminance step between outdoors and indoors is significantly reduced and, ideally, fits the photoreceptor's functional range. Eventually, no adaptation time is needed to perceive low contrast objects, e.g., a tripping hazard, and the visual system can be sufficiently supported during an indoor-outdoor transition. Currently, two different technical approaches of self-tinting lenses exist: photochromic [15,16] and electrochromic lenses [17,18]. The tinting process of photochromic lenses relays on a photochemical reaction were ultraviolet light leads to photoisomerization of for example, microcrystalline silver halides resulting in a visible color and transmission change of the lens [19]. However, photochromic lenses come with a few drawbacks. First, because their reaction mainly depends on ultraviolet light, photochromic lenses do not tint behind a window or a car's windshield. Secondly, the photoisomerization process needs on average 210 s to switch between states at room temperature [20], which is too slow for most of the encountered indoor-outdoor transitions. The latest solutions are ultraviolet-light independent electrochromic lenses. Here, the tinting is the result of an electrochemical reaction of for example tungsten trioxide to an electric stimulus [21]. Recently, a broad variability of electrochromic materials has emerged with highly variable characteristics regarding their switching times and transmission values [22,23]. To this point it is unclear which electrochromic lens characteristics (e.g., the available transmission range, minimum and maximum transmission, or response time), are physiologically relevant or - in case of a trade-off - more important to support the visual system enabling optimal vision in modern life.

We here introduce a virtual-reality (VR) based simulation platform to study visual performance in the context of indoor-outdoor transitions and the benefit of self-tinting lenses. For this purpose, at first the VR headset was characterized to assess its potential to mimic the encountered illumination changes of indoor-outdoor transitions. Followed by setting up a VR environment to simulate such transitions as well as the respective transmission characteristics of self-tinting lenses. Lastly, to obtain an objective read-out of visual performance after a transition, a test for reaction times to detect low contrast targets was implemented. Based on the hypothesis that a self-tinting lens ideally supports the visual system by overcoming the need for luminance adaptation, reduced detection times in cases with self-tinting lenses compared to static transmission lenses should be observed.

## 2 Results

### 2.1 Linearization

**Fig. 1:**
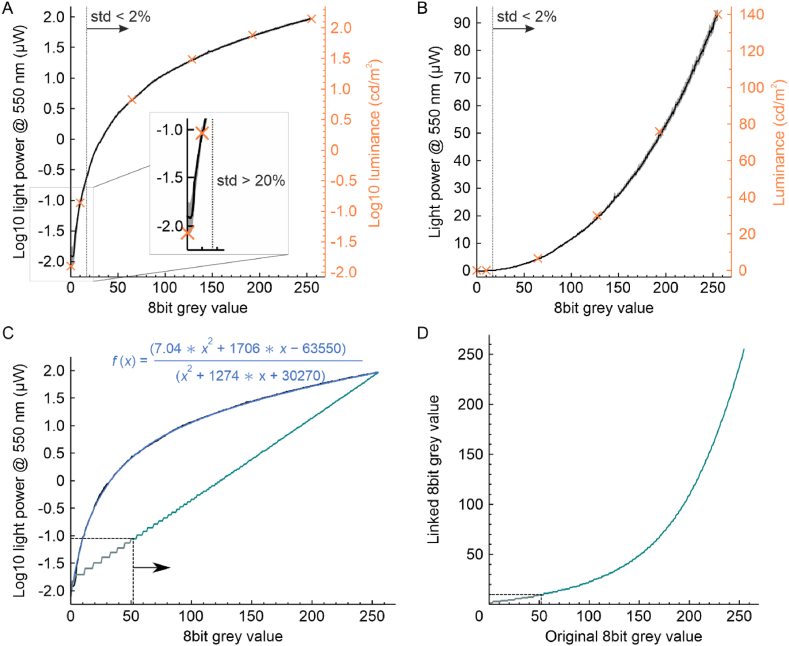
Output power linearization for the HTC Vive HMD. A & B) Set grey level and corresponding average logarithmic or linear scaled output power in μW. The grey shaded area shows ±1 standard deviation of repeated measurements. For grey values ≥ 17 the standard deviation for repeated measures was <2 %. For grey values < 8 standard deviation was larger than 20 %, highlighted by the inset. Luminance was measured at selected data points (orange crosses), confirming the validity of the used power values for linearization. C) The average logarithmic power values were fitted with a rational function (blue, equation stated). 8bit grey values were linked according to D). From the linearized output (turquoise line in C), only 8bit grey values ≥ 10 were used leaving a residual luminance range of 3 magnitudes. (For interpretation of the references to color in this figure legend, the reader is referred to the Web version of this article.)

Linearization of the used HTC Vive VR headset's output luminance was achieved by using a shader to directly apply an achromatic 8bit RGB value (in the following referred to as grey value) to a surface covering the entire display. The use of a custom shader ensured full control of the surface's ultimately rendered grey value. Firstly, at each grey value (in the range 0–255), the resulting light power was measured repeatedly three times while continuously increasing or decreasing grey values (see Fig. 1). Repeated power measurements showed a relative standard deviation of about 2 % for grey values between 17 and 255. For low grey values (grey values between 8 and 16), the relative measurement variability was about 5 % and for the residual lowest grey values (0–7) measurement variability was about 20 %. Secondly, at 255, 0, and 4 intermediate grey levels the luminance was measured, confirming a direct relationship between power meter readings and actual luminance (see Table 1). Repeated luminance measurements showed a variability of about 3 %. At grey value 0 a residual power of approximately 13 nW and luminance of 0.014 cd/m^2^ were measured on average. At 255 the power was about 93 μW and luminance was 140 cd/m^2^ yielding a maximum contrast ratio of approximately 1:10,000 or a possible luminance range of 4 magnitudes.

**Table 1:** Comparative listing of the HMD's average output power and luminance measurements.

Grey value	Power meter reading [μW]	Log10 (power)	Contrast ratio	Luminance [cd/m^2^]	Log10 (lum.)	Contrast ratio
255	93	1.97	1.0	140	2.15	1.0
192	48	1.68	1.9	76	1.88	1.8
128	19	1.29	4.8	30	1.48	4.7
64	4.3	0.63	22	6.6	0.82	21
10	0.095	−1.02	979	0.14	−0.85	1000
0	0.013	−1.89	7152	0.013	−1.89	10769

The relationship between grey values and their average output power was fitted with a rational function (Fig. 1C):

$$f\left(x\right)=\frac{\left(7.04*x^{2}+1706*x-63550\right)}{\left(x^{2}+1274*x+30270\right)}$$

Using this fit, linearization was achieved by creating a look-up-table linking each grey value between 0 and 255 to a new grey value (Fig. 1D) enabling equal changes in luminance on a logarithmic scale. The logarithmic scale was chosen due to “Fechner-Law” stating that the perception of luminance scales proportionally to logarithmic changes, meaning that for a sensation of linear luminance changes, logarithmic changes are required [24]. In Unity, the linearization was realized based on the “Dictionary” class to link the respective grey value pairs in a look-up-table. For the final transition simulation, grey values from 10 to 255 were used, because of the high measurement uncertainty for low grey values and relatively large luminance increments between single steps for grey values < 10. According to Table 1, the lower grey value of 10 corresponds to a luminance of 0.14 cd/m^2^, while the upper grey value of 255 corresponds to 140 cd/m^2^. Thus, the selected grey value range enabled a range of 3 magnitudes for luminance changes.

### 2.2 Transmission implementation

This look-up-table was also used for the calculations to convert any required transmission, of a currently simulated spectacle lens, into the respective alpha value. To this end, it was mandatory to set the project's color space to “gamma” [25]. At the beginning of each frame update the luminance value behind the lens “Lum_behind_” is calculated as the product of the current wall luminance “Lum_original_” and the transmission of the simulated lens:

$${Lum}_{behind}={Lum}_{original}*Transmission$$

The respective 8bit grey value (“8bitGrey_behind_”) was determined by using the look-up-table in reverse (method “FirstOrDefault”) and finding the closest entry for the needed modified luminance “Lum_behind_”. The simulated lens’ alpha could then be calculated as

$${Alpha}_{lens}=1-\left(\frac{{8bitGrey}_{behind}}{{8bitGrey}_{original}}\right)$$

and was applied to the canvas simulating the lens.

**Fig. 2:**
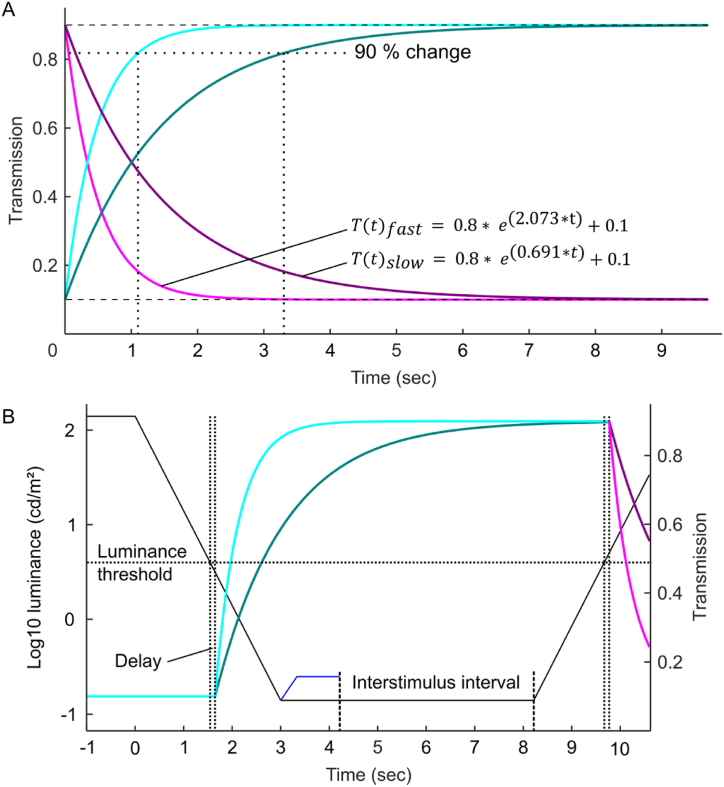
The self-tinting lenses' transmission change over time is given by an exponential function. A) Transmission changes for the fast and slow self-tinting. The respective response times, given by a 90 % completed change (dotted lines), are about 1 and 3 s. B) Complete illustration of the self-tinting lens assessment protocol. Each trial starts with a luminance change across several magnitudes. Crossing the luminance threshold triggers the transmission change after a set delay (here 100 msec). The stimulus fades in when the target luminance is reached and is deleted on detection. Before the next trial starts, the interstimulus interval allows the visual system to adapt to the new lighting.

While normal spectacle lenses or sunglasses have a static transmission, self-tinting (such as electrochromic or photochromic) lenses are designed to have a dynamic transmission. This dynamic transmission was implemented with a linear as well as a more realistic exponential switching behavior. The simpler linear switching allowed a direct and therefore easier control of maximum and minimum transmission, and response time. The more complex, but realistic exponential switching was implemented by using MATLAB to compute an exponential function according to the desired specification. Here, two different self-tinting lenses were simulated. Both had a similar range of transmission states, with a maximum transmission of 90 % and a minimum transmission of 10 %. The “fast” switching self-tinting lens had a response time (defined by reaching 90 % of the target transmission) of about 1 s, and the “slow” switching had a response time of approximately 3 s (see Fig. 2A). The resulting exponential functions describing the switching from clear to dark were:

$${T\left(t\right)}_{fast}=0.8*\;e^{\left(2.073*t\right)}+0.1$$

$${T\left(t\right)}_{slow}=0.8*\;e^{\left(0.691*t\right)}+0.1$$

And for switching from dark to clear:

$${T\left(t\right)}_{fast}=-0.8*\;e^{\left(2.073*t\right)}+0.9$$

$${T\left(t\right)}_{slow}=-0.8*\;e^{\left(0.691*t\right)}+0.9$$

In the following psychophysical assessment procedure for testing visual performance, transmission changes of self-tinting lenses were automatically triggered by a virtual binary light sensor with a threshold of 4 cd/m^2^ (8bit grey = 54). Between the light sensor switch and the onset of the transmission, a simulated electronic delay of 100 msec was added (Fig. 2B).

### 2.3 Psychophysical assessment

**Fig. 3:**
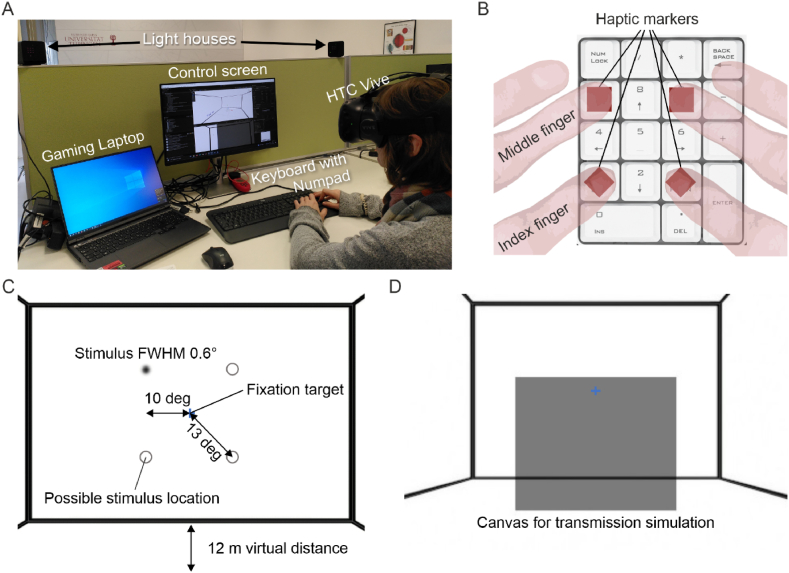
Psychophysical assessment of visual performance after steep luminance changes using virtual reality. A) Real-world environment and hardware used for the study. B) Participants had to respond in which corner the stimulus appeared via a numpad. Because this was not visible when wearing the headset, the corresponding keys were marked with tape to provide haptic feedback. Additionally, participants were asked to place their fingers on the numpad like shown in the figure, to avoid wrong key presses. C) The VR scenery rendered via Unity with the relevant spatial stimulus parameters highlighted. D) A screenshot from the participant's point of view with the canvas simulating any given lens transmission rate.

The psychophysical assessment procedure of visual performance consisted of 40 trials for each of the 8 different conditions (see Table 2), with increasing and decreasing lighting scenarios tested consecutively. Because of the logarithmic linearization, the luminance could be changed at a constant rate of 1 log10 units per second (Fig. 2B) or 3 log10 units/sec by setting the grey value of the walls, which acted as the light source of the scene, through Unity. When the target luminance was reached the stimulus faded in at 0.3 log10 units/sec. The starting luminance of the stimulus was adjusted to ensure a fixed fade-in time of 333 msec. The target luminance was empirically determined to make sure that stimuli were only just perceivable, resulting in unequal Weber-contrast values for the sunglasses mode (2.5) compared to normal or self-tinting lenses (0.7) and between luminance increases and decreases (see Table 3, Table 4). Weber-contrast was similar across conditions with about −0.2 at high luminance. The stimulus appeared randomly in one of four corners (Fig. 3). Participants (see STAR methods for details) pressed the corresponding key on the numpad on stimulus detection, for example, “7” for the upper left corner. When the location was correctly reported, the stimulus object was deleted. Before the next trial was initialized an interstimulus interval of 4 s had to be awaited allowing the visual system to adapt to the new light condition. In the following, detection times are stated as Median and interquartile range (IQR) in seconds. The statistical details are given in the STAR methods.

**Table 2:**
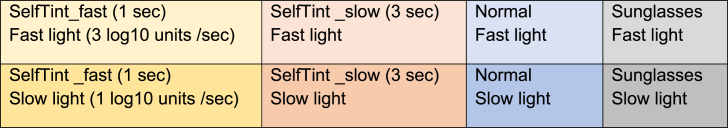
Test scenarios for psychophysical assessment of visual performance with and without self-tinting lenses (“SelfTint”). Each scenario included 40 trials in total (20 trials for room lights switching off and 20 trials for switching on). A detailed description of each lens type is given in the main text.

**Table 3:** Weber contrast for stimuli at low light conditions.

	8bit grey (original)	Transmission/Alpha	8bit grey (lens)	Luminance (cd/m^2^)	Weber contrast
Normal (Wall)	10	0.95/0	10	0.14	0.71
Normal (Stimulus)	14		14	0.24	
Sunglasses (Wall)	10	0.20/0.8	2	0.02	2.50
Sunglasses (Stim)	37		7	0.07	
SelfTint (Wall)	10	0.70/0.2	8	0.09	0.67
SelfTint (Stimulus)	14		11	0.15

**Table 4:** Weber contrast for stimuli at bright light conditions.

	8bit grey (original)	Transmission/Alpha	8bit grey (lens)	Luminance (cd/m^2^)	Weber contrast
Normal (Wall)	255	0.95/0.016	251	135	−0.22
Normal (Stimulus)	230		226	105	
Sunglasses (Wall)	255	0.20/0.506	126	28	−0.21
Sunglasses (Stim)	230		113	22	
SelfTint (Wall)	255	0.30/0.408	151	41	−0.20
SelfTint (Stimulus)	230		136	33

**Fig. 4:**
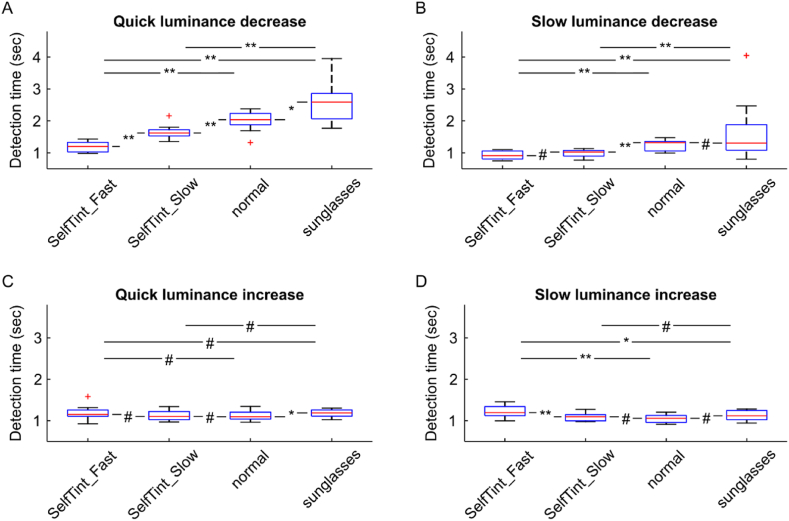
Detection times after luminance changes for all different lens conditions. A) and B) show detection times after quick (3 log10 units/sec) and slow (1 log10 unit/sec) luminance decreases. C) and D) show detection times after luminance increases. Asterisks denote significance level (#p > 0.05; ∗p < 0.05; ∗∗p < 0.01; N = 10).

After a quick luminance decrease, detection times were significantly lower in the fast self-tinting condition (1.20 (0.30), all p < 0.01; Fig. 4A). Detection times increased for the slow self-tinting lens (1.62 (0.19)) but were significantly lower (all p < 0.01) than in normal (2.04 (0.36)) or sunglasses mode (2.59 (0.79)). The observed increase in detection times for sunglasses compared to normal lenses was significant as well (p < 0.05). After a slow luminance decrease, again detection times were lowest for the fast self-tinting lens (0.91 (0.25); Fig. 4B), followed by the slow self-tinting lens (1.02 (0.18)). This difference in detection times between the two self-tinting lens modes was not significant. However, significantly higher detection times were observed for the static transmission lens modes (all p < 0.01). For normal and sunglasses lens mode detection times were similar and not significantly different (1.32 (0.30) and 1.31 (0.80), respectively). When luminance was increased by 3 magnitudes detection times were similar across different lens conditions and the two different change rates (for 3 log10 units/sec: 1.15 (0.16), 1.10 (0.20), 1.09 (0.17), 1.19 (0.15); for 1 log units/sec: 1.20 (0.22), 1.09 (0.15), 1.06 (0.17), 1.12 (0.23) for fast and slow self-tinting, normal and sunglasses respectively; Fig. 4C and D). Detection times were significantly higher for simulated sunglasses compared to normal lenses after quick luminance increases (p < 0.05) and significantly higher for the fast self-tinting lenses compared to all other lens modes after slow light increases (p < 0.01 and p < 0.05).

For repeated reaction time recordings within a given test condition an average standard deviation of 295 msec was observed. Additionally, reliability of the recorded data was analyzed based on the observation of stable and condition independent reaction times after luminance increases. Reaction times of the last 20 trials were significantly shorter by 70 msec compared to the initial 20 trials (RT_1-20_ = 1.23 ± 0.08 s and RT_140-160_ = 1.16 ± 0.06 s; p = 0.003; one-sided, unpaired *t*-test; data not shown).

## 3 Discussion

We used an HTC Vive virtual reality headset to create a simulation platform for studying human visual perception during significant luminance changes (≥3 magnitudes), such as those experienced during indoor-outdoor transitions (e.g., driving into a tunnel). Since the photoreceptors can only cover changes of up to 1.5 log10 units, adaptation is necessary. During this adaptation period, humans are unable to perceive low-contrast objects, like a small obstacle, which could be a tripping hazard. Therefore, indoor-outdoor transitions elicit a high level of visual discomfort, which can also be objectively assessed by for example measuring fluctuations in pupil diameter [13]. Self-tinting lenses could potentially assist in these situations by modulating their transmission to reduce the encountered luminance change. However, the required characteristics of a self-tinting spectacle to make for a sufficient aid by optimally supporting visual perception are unclear. To this end, a simulation of transmission changing spectacle lenses was added to the indoor-outdoor transition simulation platform in combination with a detection time assessment. Because the ideal self-tinting lens should obviate the otherwise needed time for adaptation, significantly decreased detection times for low contrast objects should be observed after a in cases of a self-tinting lens.

Although the HTC Vive headset could not display the exact luminance values of outdoor scenes, its luminance range after linearization allowed us to simulate luminance changes up to 3 magnitudes. This is sufficient to replicate scenarios like driving into a tunnel (from 5000 cd/m^2^ to 4 cd/m^2^) [9,26] or entering a building on a sunny day (a drop from about 4 to 2 log10 units) [27,28].

Detection times observed here, were consistent across conditions after luminance increases (on average 1.1 s). However, for luminance decreases, detection times varied significantly. between the tested conditions. Trials with simulated self-tinting lenses, and therefore dynamic transmission rates, had the lowest detection times of about 1.2 s on average across conditions followed by normal static transmission lenses (2.0 and 1.3 s for quick and slow luminance changes, respectively) and simulated sunglasses (2.6 and 1.3). This observed difference in detection times, being significantly shorter in case of a lens with a dynamic transmission compared to a static transmission (“normal” and “sunglasses”), proves the initial hypothesis that self-tinting can successfully preserve optimal visual performance during large luminance changes. This is most likely achieved by reducing the range of the encountered luminance change and thereby circumvents the need for adaptation as it is the case for lenses with static transmission resulting in elevated reaction times. Additionally, it could be demonstrated, that such benefit of self-tinting lenses strongly depends on the time needed to change transmission and the slope of the overall luminance change.

These observed detection times are much higher compared to simple reaction times of about 400 msec reported for peripheral testing with mesopic luminance [29,30]. There are several reasons that could explain the large difference between our study and previous reports. First, the stimulus used was a blob with a full width at half maximum (FWHM) of 0.6° and thereby up to three times smaller than the 1.5–2° diameter circular stimuli used in other psychophysical experiments [26,29,30]. Second, being a blob, the stimulus did not have a distinct grey level step at the edges, to make detection more challenging. Third, the stimulus did not suddenly appear, but contrast increased during the first 330 msec of the presentation time to make detection even more challenging. This is also reflected in the fact that when neglecting the 330 msec fade-in time, the observed detection times are close to reported search reaction times of about 750 msec for a small set size [31].

Given this more demanding task, a relatively high standard deviation of 295 msec on average seems reasonable and does not reduce the reliability in the stated findings. Because the observed changes in reaction times (e.g.: 420, 380, and 550 msec for significant median shifts after quick luminance changes) were thusly larger than the measurement variability, and a sufficient signal-to-noise ratio can be assumed for the test method.

The duration of the experiment was about 1 h, which could have led to fatigue and therefore increasing reaction times throughout the experiment affecting later reaction time recordings. For this reason, the sequence of test conditions was randomized for each participant. Additionally, the rather stable reaction times of the first and last 20 trials after luminance increases were compared and revealed a significant decrease of about 70 msec. This means that in total learning effects were stronger than fatigue or habituation. However, compared to the overall variability, given a standard deviation of 295 msec, this absolute shift in reaction times can be neglected.

Because stimulus presentation time was not limited to a short presentation time and participants were allowed to look freely, the stimulus location on the retina was unclear. But at least simple reaction times for central and peripheral stimuli (7.5° eccentricity, 0.8° diameter) are similar at about 300 msec [32].

Interestingly, detection times were similar for both increasing and decreasing luminance in the self-tinting lens condition, despite different stimulus contrasts (about −0.2 and 0.7, respectively). This aligns with prior studies, reporting increasing simple reaction times for decreasing background luminance but the same Weber-contrast of small peripheral (10° eccentricity) stimuli [29,30,33]. Or vice versa, to achieve similar reaction times at 1 cd/m^2^ and 0.1 cd/m^2^ background luminance, the Weber contrast needed to be significantly increased from 0.2 to 0.7 [29]. At 0.01 cd/m^2^ (comparable to our sunglasses condition; Table 3), even with a contrast of 0.8, reaction times were 100 msec slower than at 0.1 cd/m^2^. Our data suggest that a contrast >3 would be required to achieve reaction times comparable with the 0.1 cd/m^2^ background luminance and 0.7 contrast condition [29]. This is our observation, that stimulus contrast for the sunglasses condition was much higher (2.5) than for the other two conditions, for similar detection times between the static transmission scenarios. Additionally, for a simple visual discrimination task under low mesopic viewing conditions it was reported that d-prime (a measure of sensitivity) decreases, too [34]. As a side note, these observations provide a scientific justification why class 4 sunglasses (transmission rate <8 %) are not suitable and forbidden for driving, due to increased reaction times in tunnels.

Detection times were slightly longer for luminance decreases compared to increases, which were consistent across conditions. This may be due to the faster adaptation to light than to darkness [35]. Dark adaptation relies on the visual cycle's metabolism to replenish photopigment, and the gap between light and dark adaptation increases with age [35]. Thus, self-tinting spectacles could be particularly beneficial for elderly individuals during indoor-outdoor transitions by reducing the need for adaptation to see well.

For future studies utilizing this simulation platform, it could be worthwhile to fade in the stimulus by increasing its size rather than increasing contrast. In this way, stimulus emergence would be closer to a real-world scenario where e.g., a driver moves closer to an obstacle. In the current version the self-tinting lens was simulated by adjusting the alpha value of an UI canvas with a fixed grey value. However, it is known that the spectral absorbance changes when switching between states [18] and should be included as well, for a more realistic simulation of a self-tinting lens. Furthermore, including pupil tracking could help to provide an objective readout for the participant's visual discomfort during rapid luminance changes, as it was shown that visual discomfort correlates with fluctuations in pupil diameter [13]. Such pupil diameter fluctuations were reported for an increased cognitive load as well [36,37].

With the available range of 4 log10 units, the HTC Vive headset could be utilized for dark adaptometry, if equipped with neutral density filters. Dark adaptometry can be useful as an early indicator of e.g., age-related macular degeneration [38] or other retinal diseases related to systemic vitamin A deficiency, where impaired dark adaptation leads to a complete dark-adapted rod sensitivity [39,40].

### 3.1 Limitations

In the shown proof-of-concept psychophysical experiment detection times for small low-contrast stimuli after steep luminance changes with a rate of 1 or 3 log10 units/sec were applied. These rates are much steeper compared to entering a tunnel with modern lighting where luminance changes stepwise by 3 log10 units within 7 s (about 0.4 log10 units/sec) [9,26]. However, to our best knowledge no literature exists on the timescale of luminance change rates when walking into a building or driving into a tunnel without modern lighting.

## 4 Conclusions

The presented simulation platform to study human visual perception related to large changes in luminance demonstrated that the available luminance output range of the used HTC Vive headset sufficiently covers the typically encountered relative range of real-world luminance changes. Therefore, VR can be a very useful tool to minimize the efforts needed to develop a new lighting or lens design to support optimal visual performance in a modern daily life. For example, when searching for an optimal lens design, all possible lens characteristics can be simulated in VR without the actual need of an operational self-tinting lens prototype. In case of tunnel lighting studies road closures and complex conversion measures can be avoided by testing a battery of possible solutions in VR first, until the ideal design is found. However, since the headset can only represent the range but not the absolute luminance values, the final design should always be assessed in a final real-world experiment. The observed detection times across the different conditions tested here are in good accordance with previous studies reporting increasing reaction times for low background luminance levels at the same stimulus contrast or similar reaction times when stimulus contrast was increased for low background luminance levels. The implementation of a self-tinting lens simulation into this simulation platform will be useful to determine the required range and time scale of transmission changes to sufficiently bolster visual perception during indoor-outdoor transitions. Future studies assessing self-tinting lens characteristics may include the usefulness of intermediate transmission states as well as the user acceptance of manual or automatic transmission control.

## CRediT authorship contribution statement

**Niklas Domdei:** Writing – review & editing, Writing – original draft, Visualization, Validation, Software, Methodology, Investigation, Formal analysis, Data curation, Conceptualization. **Yannick Sauer:** Writing – review & editing, Software. **Brian Hecox:** Writing – review & editing, Software. **Alexander Neugebauer:** Writing – review & editing, Software. **Siegfried Wahl:** Writing – review & editing, Supervision, Resources, Funding acquisition, Conceptualization.

## Data and code availability

Data from this experimental study have been deposited at Mendeley and are publicly available as of the date of publication. The DOI is listed in the key resources table.

All original code has been deposited at github and is publicly available as of the date of publication. The URL is listed in the key resources table.

Any additional information required to reanalyze the data reported in this paper is available from the lead contact upon request.

## Declaration of competing interest

The authors declare the following financial interests/personal relationships which may be considered as potential competing interests: Siegfried Wahl reports a relationship with Carl Zeiss Vision International GmbH that includes: employment. Co-authors Niklas Domdei and Yannick Sauer are employed by Carl Zeiss Vision International GmbH. Co-author Brian Hecox is employed by Bad Ridge Games, Seattle, Washington, USA. There are no conflicts of interests regarding this study. The funders did not have any additional role in the study design, data collection and analysis, decision to publish or preparation of the manuscript.If there are other authors, they declare that they have no known competing financial interests or personal relationships that could have appeared to influence the work reported in this paper.The paper is pre-published on https://www.biorxiv.org/content/10.1101/2024.04.16.589684v1.

## Figures and Tables

**Fig. 5:**
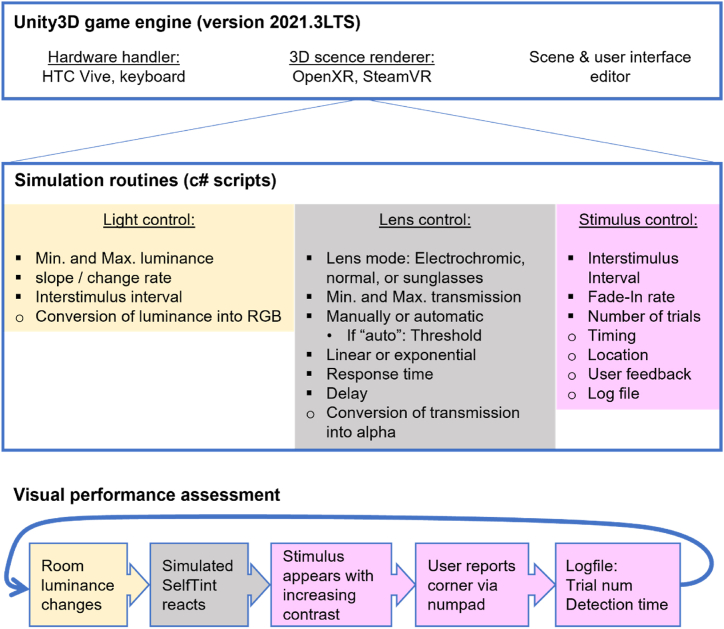
Basic software architecture and psychophysical assessment logic of the proposed simulation platform. Public variables that can be modified through the unity interface by an operator are marked with filled squares. Open circles denote routines embedded into the c#-scripts that cannot be modified externally.

## Star Methods

### Lead contact

Further information and requests for resources should be directed to and will be fulfilled by the lead contact, Siegfried Wahl (siegfried.wahl@uni-tuebingen.de).

### Experimental model and study participant details

#### Study participants

For the psychophysical evaluation procedure, a total of 10 healthy participants (4 males; mean age 29 ± 4 years) were recruited in this study, all members of the workgroup at the University of Tübingen. All participants had normal or corrected-to-normal vision, with no known retinal pathologies.

The study adhered to the tenets of the Declaration of Helsinki. The ethics authorization to perform the measurements was granted by the Medicine Faculty Human Research Ethics Committee from the University of Tübingen. Prior to data collection, the experiment was explained in detail to the participants, and written informed consent was collected from each participant. All data were pseudonymized and stored in full compliance with the principles of the Data Protection Act GDPR 2016/679 of the European Union.

### Method details

#### Hardware

Based on a preliminary study from our lab, it was decided to use the HTC Vive (HTC Cooperation, Taoyuan, Taiwan) VR head-mounted display (HMD) as it provides the largest range of luminance levels known for HMDs [41], due to its organic light-emitting diode (OLED) display technology. The individually specific output light power levels of the used HMD were assessed with a power meter (818-SL/DB sensor with 843-R power meter, Newport, USA) and a self-written Unity3D (version 2021.3.12f1; Unity Technologies, CA, USA) environment setting the HMD's OLED displays to a given achromatic grey level between 0 and 255. For the linearization measurements, a central wavelength of 550 nm was set in the power meter settings. The sensor was placed directly behind the right eye's lens and sensor as well as headset covered with black fabric to block any outside light. Successful elimination of any outside light leakage was confirmed via a light power measurement when the headset was switched off. Additionally, a spot luminance meter (LS-110, Konica Minolta, Inc., Tokyo, Japan) with a close-up lens was used to ensure the measured range and to link the measured light power values with actual luminance values [25]. Here again, the luminance meter was placed directly on the headset's lens. Luminance uniformity was confirmed firstly by displacing the photo sensor respective to the lens center showing small fluctuations (<5 %) and secondly via comparison of the close-up lens measurement with a full field measurement placing the luminance meter at 50 cm distance revealing only a minor difference (<10 %).

#### Software

The indoor-outdoor transition simulation was set up as a Unity3D scene using separated c#-scripts to control the different features of the simulation platform (Fig. 5). To ensure that the pixel values of the walls can be set directly and are not influenced by light effects, the shader of the wall's material was set to “unlit” from the universal render pipeline, rendering the objects, or here the walls, without any lighting effects. This way the overall VR room lighting could be controlled by accessing and modifying the wall's material color specifications. The self-tinting lenses were simulated with a UI canvas attached to the main camera at a 0.1 m virtual distance. The specified transmission was achieved by accessing and modifying the canvas' alpha specifications. For the conversion between transmission and alpha values the project's color space was set to “gamma” [25]. Additionally to the implications mentioned by Murray et al., clipping artifacts were observed when set to “linear” color space, where dark grey values were rendered as black thereby ruling out a realistic transmission simulation.

The VR presentation of the unity scene on the HMD was enabled through the OpenXR packages in combination with the SteamVR (Valve Cooperation, Bellevue, WA, USA) plug-in.

#### Psychophysical procedure

At the beginning of the experiment the participant put on the headset and was asked to adjust the straps of the headset for an optimal fit to ensure a clear view of scene through the center of the headset's Fresnel lenses. This whole procedure took roughly 1 Minute. During this time the light in the virtual scene was always on. The general task for the participants was to detect a small achromatic low-contrast blob stimulus, appearing in one of four corners (top left, top right, bottom left, bottom right) at a 12 m virtual distance, and report the correct corner via pressing the corresponding key on a numpad (Fig. 2). The four possible stimulus locations were displaced from a central fixation target by about 13°. The FWHM of the stimulus was about 0.6°. The stimulus material used the “unlit” shader of the universal render pipeline, and the stimulus contrast was controlled by adjusting the material's color specifications.

To assess visual performance after rapid luminance changes, participants had to detect the stimuli in a total of eight different scenarios, with 4 different lens and 2 luminance change rate settings (see Table 2). Luminance changed in the “fast” setting with a constant rate of 3 log10 units/sec and 1 log10 unit/sec for “slow” by controlling the grey value of the walls, acting as light sources of the scene, via Unity based on the calibrated look-up-table. The 4 different lens types, simulated by controlling the transparency of a canvas in front of the virtual camera, were characterized by their respective transmission rates. For the self-tinting lenses, transmission was dynamic with 2 different response times defined by reaching 90 % of the target transmission value. The “quick” lens had a response time of 1 s the “slow” on of 1 s. Additionally, 2 lenses with static transmission were simulated, acting as a control condition. The “normal” lens had a static transmission of 95 % and the “sunglasses” 20 % transmission.

Each condition consisted of 40 trials in total with 20 trials for room lights switching off and 20 trials for switching on. On stimulus detection, participants had to press the respective key on a numpad, and the reaction time needed for detection was recorded. Between individual trials, a brief interval of 4 s was added, until the next light change was initialized (see Fig. 2B). The duration for this interval was chosen based on a similar real-world experiment, reporting a maximum of 2 s for young participants to dark adapt, after a luminance decrease of 1.2 log10 units. Thus, for the here applied luminance change of 3 log10 units (0.14 cd/m^2^ to 140 cd/m^2^), 5 s would be required. This was achieved by adding an interstimulus interval of 4 s after stimulus detection, which was determined to take at least 1 s for the given task.

Participants underwent two training runs (80 trials in total) prior to the actual testing runs in the “Normal” condition. The different conditions were tested in randomized order to minimize any influence from further training of the task or fatigue during the ongoing experiment. The individual trial, starting with a change in luminance and ending with the participant's response was on average 4.0 ± 1.0 s. The average run duration was about 5.3 ± 0.6 min, and the whole experiment (8 runs + 2 training runs) took about 60 min on average.

#### Quantification and statistical analysis

The statistical analysis of the recorded detection times was done in MATLAB (The MathWorks, Inc., Natick, USA). First, outliers were identified as being more than 1.5 times the interquartile range higher than the 3rd quartile or lower than the 1st quartile and were removed. On average approximately 0.7 outliers per participant per condition occurred (minimum 0 – maximum 5 times), with a total of 115 outliers out of all 3200 trials recorded (3.6 %). Second, the participant's median detection times were compared using the Wilcoxon signed rank test (Matlab: “signrank”), as data sets were not normally distributed (Kolmogorov-Smirnov test; Matlab: “kstest”).
